# Identification of *Felis catus* Gammaherpesvirus 1 in Tsushima Leopard Cats (*Prionailurus bengalensis euptilurus*) on Tsushima Island, Japan

**DOI:** 10.3390/v10070378

**Published:** 2018-07-19

**Authors:** Isaac Makundi, Yushi Koshida, Yasuyuki Endo, Kazuo Nishigaki

**Affiliations:** 1 The United Graduate School of Veterinary Science, Yamaguchi University, 1677-1 Yoshida, Yamaguchi 753-8515, Japan; isaac.makundi@sua.ac.tz; 2 College of Veterinary Medicine and Biomedical Sciences, Sokoine University of Agriculture, P.O. BOX 3019, Morogoro 67125, Tanzania; 3 Conservation and Animal Welfare Trust, Tsushima, 642-2 Kamiagata, Tsushima, Nagasaki 817-1602, Japan; koshida98044@yahoo.co.jp; 4 Joint Faculty of Veterinary Medicine, Kagoshima University, 1-21-24 Kagoshima, Kagoshima 890-0065, Japan; k5155981@kadai.jp

**Keywords:** gammaherpesvirus, feline, domestic cat, Tsushima leopard cat, Tsushima Island, FcaGHV1, molecular epidemiology

## Abstract

*Felis catus* gammaherpesvirus 1 (FcaGHV1) is a widely endemic infection of domestic cats. Current epidemiological data identify domestic cats as the sole natural host for FcaGHV1. The Tsushima leopard cat (TLC; *Prionailurus bengalensis euptilurus)* is a critically endangered species that lives only on Tsushima Island, Nagasaki, Japan. Nested PCR was used to test the blood or spleen of 89 TLCs for FcaGHV1 DNA; three (3.37%; 95% CI, 0.70–9.54) were positive. For TLC management purposes, we also screened domestic cats and the virus was detected in 13.02% (95% CI, 8.83–18.27) of 215 cats. Regarding phylogeny, the partial sequences of FcaGHV1 from domestic cats and TLCs formed one cluster, indicating that similar strains circulate in both populations. In domestic cats, we found no significant difference in FcaGHV1 detection in feline immunodeficiency virus-infected (*p* = 0.080) or feline leukemia virus-infected (*p* = 0.163) cats, but males were significantly more likely to be FcaGHV1 positive (odds ratio, 5.86; 95% CI, 2.27–15.14) than females. The higher frequency of FcaGHV1 detection in domestic cats than TLCs, and the location of the viral DNA sequences from both cats within the same genetic cluster suggests that virus transmission from domestic cats to TLCs is likely.

## 1. Introduction

The Tsushima leopard cat (TLC: *Prionailurus bengalensis euptilurus*; family Felidae) is a small wild cat inhabiting Tsushima Island, Nagasaki, Japan. Most TLCs live in Kamijima but a few live in Shimojima. The TLC has been designated a critically endangered species, and several management strategies have been implemented to maintain the species [1,2]. Interspecies transmission of several diseases from free-ranging domestic cats to TLCs have been reported [1,3,4,5]. Feline immunodeficiency virus (FIV) isolated from a wild TLC shared *env* gene sequences with FIV isolated from domestic cats [5]. In addition, a study that examined FIV infection risk in TLCs using Geographical Information System (GIS) data found that TLCs living in areas densely populated with domestic cats were at higher risk of infection than those from areas with fewer domestic cats [4]. We recently reported the prevalence of feline leukemia virus (FeLV) infection in domestic cats on Tsushima Island. We did not detect FeLV in TLCs; however, we demonstrated that the virus could replicate in their cells [6].

Gammaherpesvirus (GHV) infection is typically characterized by an extended period of viral latency in the host that persists for the host’s lifetime [7,8]. There is cumulative evidence of GHV infection in several felid species [9,10,11,12], suggesting that many animal species are infected with one or more GHVs [13]. For example, two GHV strains, LruGHV1 and LruGHV2, have been isolated from bobcats (*Lynx rufus*). In addition, two different GHV strains, PcoGHV1 and LruGHV1, have been isolated from pumas (*Puma concolor*) [12].

*Felis catus* gammaherpesvirus 1 (FcaGHV1) commonly infects domestic cats and has a worldwide distribution [14,15,16,17]. To date, FcaGHV1 DNA has not been detected in other feline species; therefore, the host range of FcaGHV1 is currently unknown [18]. Previous reports support the pathogenic potential of FcaGHV1 infection as FcaGHV1-positive cats were at least twice more likely to be in ill-health than healthy on physical examination [14,16]. The disease outcome and risks associated with the transmission of FcaGHV1 from domestic cats to TLCs is currently unknown. Therefore, monitoring the TLC population for infectious diseases is highly recommended as part of a surveillance and management strategy since measures required to control disease in wild populations can be challenging [6]. The expansion of human habitats facilitates the spill-over of feline pathogens from domestic cats into wildlife populations; thus, determination of the pathogenic potential of FcaGHV1 is a priority for feline, human and wildlife health [14]. Previous studies have identified several risk factors for FcaGHV1 infection including adult and male status, geographical location, health status (sick) and co-pathogens [14,15,16,17,19,20,21].

The TLC usually has a large home range during breeding season and thus the possibility of contact between TLCs and free-roaming domestic cats is reasonably high [4]. The prevalence of FIV in up to 27% of the domestic cats on Tsushima Island (for example, in Kamiagata) is higher than in other regions of Japan [4]. Previous epidemiological data suggests that territorial aggression and fighting are the most common modes of FcaGHV1 and FIV transmission. Based on this association between FcaGHV1 and FIV, and on the previously reported detection of FIV in TLCs, we hypothesized that TLCs are similarly at a high risk of GHV infection.

To test this hypothesis, we designed FcaGHV1 virus-specific primers to amplify a portion of the glycoprotein B (*gB*) gene. Using these primers, we tested blood and spleen samples from TLCs and blood samples from domestic cats on Tsushima Island for FcaGHV1 DNA. We found FcaGHV1 DNA more frequently in domestic cats than in TLCs. The FcaGHV1 sequences from the TLCs were identical to some of the sequences from domestic cats over a 553-bp region of the virus.

## 2. Materials and Methods

### 2.1. Ethics Statement

This study was approved by the Institutional Animal Care and Use Committee of Yamaguchi University (identification code 2017/315, approved on 9 May 2017). Animal studies were conducted following the guidelines for the Care and Use of the Laboratory Animals of the Ministry of Education, Culture, Sports, Science and Technology, Japan.

### 2.2. Study Area

**Figure 1:**
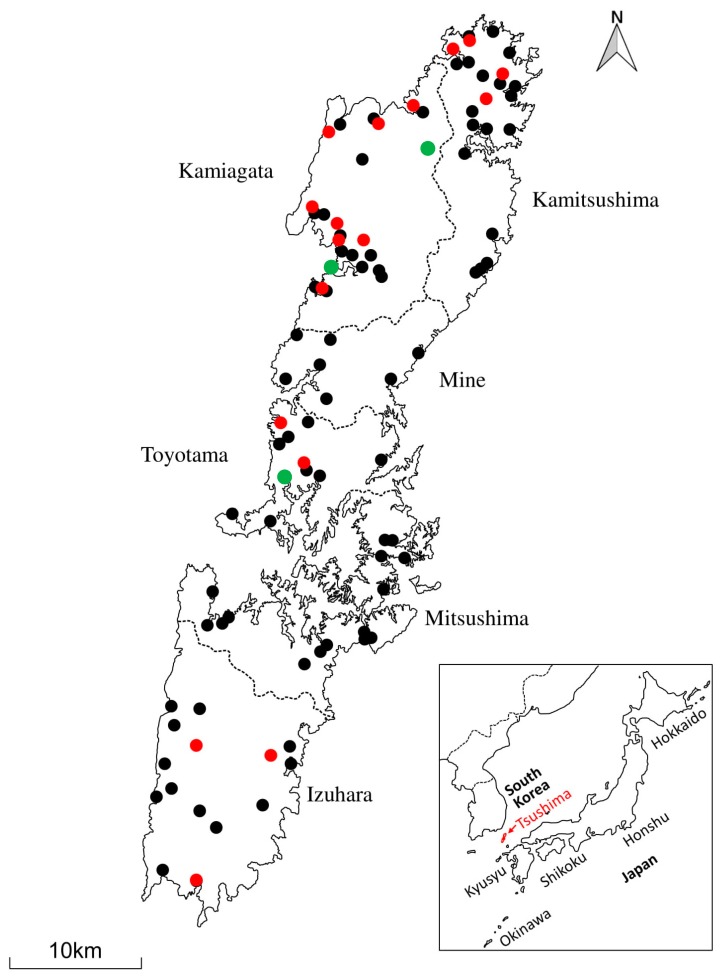
Map of Tsushima Island and sample collection sites. Black dots indicate the sites where blood sampling of domestic cats was performed. The locations of FcaGHV1-positive TLCs are shown by green dots. Sites where FcaGHV1-positive cats were detected are shown in red. The location of Tsushima Island is shown in the box.

Tsushima Island is part of the Japanese archipelago. It is situated in the northern Tsushima Strait between Japan and the Korean Peninsula. Tsushima Island actually comprises two main islands: Kamijima (north Tsushima) and Shimojima (south Tsushima). Kamijima is composed of four boroughs (Kamitsushima, Kamiagata, Mine and Toyotama) while Shimojima has two boroughs (Mitsushima and Izuhara); as shown in Figure 1.

### 2.3. Sample Collection and DNA Preparation

We collected blood and spleen samples from TLCs between 1999 and 2017. The majority of these were from animals killed by vehicles. In total, 89 TLCs (60 blood and 29 spleen) samples were available for this study [6].

Blood samples from domestic cats were donated by the Tsushima Animal Medical Center, a nonprofit animal hospital on the island. The domestic cats included both indoor-only and free-roaming cats brought by their owners to the center between 2009 and 2016 [6].

Blood samples were screened for FeLV and FIV infection using the SNAP FeLV/FIV Combo Kit (IDEXX Laboratories Inc., Westbrook, ME, USA). Chromosomal DNA from whole blood samples was purified using the Dr. GenTLE System (Takara Bio Inc., Kyoto, Japan) and DNAzol reagent (Life Technologies Japan, Tokyo, Japan) according to the manufacturer’s instructions. DNA from spleen was extracted using commercial kit (DNeasy^®^ Blood & Tissue Kit, QIAGEN, Hilden, Germany).

### 2.4. GAPDH PCR

To confirm the presence of amplifiable template DNA, a conventional PCR for feline glyceraldehyde-3-phosphate dehydrogenase (*GAPDH*) was performed as previously described [14]. Briefly, primers GAPfwd and GAPrev, designed to amplify an 80-bp product, were used. Reaction mixtures for PCR contained 0.5 µM of each primer, 2.5 mM of dNTPs, 2.5 units of TaKaRa Ex Taq DNA polymerase, and template containing up to 100 ng of DNA in a total volume of 25 µL. PCR conditions were pre-denaturation at 94 °C for 2 min followed by 30 cycles of denaturation at 94 °C for 30 s, annealing at 55 °C for 30 s and extension at 72 °C for 30 s and a final extension at 72 °C for 5 min. Electrophoresis was performed on 2.0% agarose gels in 1% Tris-acetate buffer containing 0.5 µg ethidium bromide/mL and the 80-bp product was visualized in 89 samples of TLC and 215 samples of domestic cats under ultraviolet illumination.

### 2.5. PCR Amplification and Sequencing

Using Primer3 Input (primer3.ut.ee) we designed FcaGHV1-specific primers to amplify a portion of the conserved *gB* gene based on the sequence from isolate KF840715 [11]. The method was optimized using samples confirmed by sequencing to amplify the target sequence. Nested PCR was performed using two primer pairs for the *gB* gene that amplify 715 and 580-bp gene products in the first and second rounds, respectively (Table 1). Second round reactions were employed to enhance the specificity and sensitivity of the PCR reaction. PCR was performed in 25 µL reactions using TaKaRa Ex Taq polymerase (Takara Bio Inc.). The first round PCR product (1 µL) was used as the template for the second round PCR. The PCR cycling conditions for both reactions were: pre-denaturation at 94 °C for 2 min followed by 45 cycles of denaturation at 94 °C for 30 s, annealing at 62 °C for 30 s, and extension at 72 °C for 30 s followed by a final extension at 72 °C for 7 min. The identity of the PCR amplification products was further verified by electrophoresis through 1% agarose gels, purification with the FastGene Gel/PCR extraction kit (Nippon Genetics Co. Ltd., Tokyo, Japan), and the second PCR products were sequenced in both directions by Fasmac Co., Ltd., Kanagawa, Japan. Sequences were visualized and analyzed by BioEdit [22] and, after removal of the primer sequence, the 553-bp *gB* sequences were compared with other GHV *gB* sequences using GeneTyx (Software Development Co., Tokyo, Japan) and NCBI Blast programs.

To further verify the detection of FcaGHV1 in TLCs, we PCR amplified the second adjacent conserved gene, DNA polymerase (*DNApol*), using specific primers from the same KF840715 isolate (Table 1). A nested PCR using TaKaRa Ex Taq polymerase was carried out similarly as for detection of the *gB* gene; however, the annealing temperatures for the first- and second-round PCRs were 55 °C and 60 °C, respectively. Purification of the PCR products, sequencing and sequence analysis were conducted as for the gB gene.

### 2.6. Determination of PCR Sensitivity

To determine the sensitivity of our nested PCR for the detection of FcaGHV1 *gB*, we performed serial dilutions of DNA from the peripheral blood mononuclear cells (PBMCs) and spleen of TLCs and the PBMCs of domestic cats, as previously described [23]. Six log serial dilutions of 1 g of DNA were added to the first PCR reactions and 1 µL of a 1:10 dilution of the first-round product was used for each second round of PCR. Second-round amplification products were electrophoresed at 125 V for 30 min through 1% agarose gels, stained with ethidium bromide, and visualized under UV transillumination.

### 2.7. Phylogenetic Analyses

The GHV partial *gB* and *DNApol* nucleotide sequences were aligned using ClustalW 1.6 and phylogenetic analysis was performed using MEGA6 [24]. Maximum likelihood (ML) phylogenetic analyses were conducted based on the Kimura 2-parameter model with all areas containing gaps being ignored. The betaherpesvirus human cytomegalovirus (HCMV; human herpesvirus 5{HHV5}) was used as an outgroup to root the tree. Bootstrap analysis was performed with 100 iterations to evaluate the stability of the tree.

### 2.8. Statistical Analyses

All statistical analyses were conducted using the Minitab Statistical program (Minitab version 18, Minitab Inc., Shanghai, China, 2018). The frequency of FcaGHV1 and its 95% exact confidence intervals were computed using the ML estimation. We performed descriptive analyses and created contingency tables of categorical variables with the outcome FcaGHV1 infection in domestic cats. The associations between these variables and FcaGHV1 infection were initially evaluated by univariable analysis using Fisher’s exact test and variables with *p* values < 0.2 were included in the final multivariable analysis. Univariable and multivariable analyses were performed using the Minitab 18 logit function. Binary logistic regression and model fit was evaluated by Hosmer–Lemeshow goodness-of-fit test.

## 3. Results

### 3.1. Frequency and Distribution of FcaGHV1 on Tsushima Island

In total, 89 (blood and spleen) samples from TLCs and 215 blood samples from domestic cats were available for FcaGHV1 testing (Table 2). Three TLCs were positive for FcaGHV1, giving an overall frequency of FcaGHV1 detection in TLCs of 3.37% (95% CI, 0.70–9.54). These three positive TLCs originated from Kamijima and comprised two adult females and one adult male (Figure 1).

For the purpose of TLC management and because the domestic cat is the primary natural host for FcaGHV1, we decided to investigate the infection status of FcaGHV1 in domestic cats. As shown in Table 2, the overall frequency of FcaGHV1 detection in domestic cats was 13.02% (95% CI, 8.83–18.27). The characteristics of the domestic cats tested in this study are presented in Table 3. The probability of FcaGHV1 infection in domestic cats did not significantly differ between the two test regions, Kamijima and Shimojima (Fisher’s exact test, *p* = 1.0 and odds ratio, 0.94: 95% CI, 0.36–2.47). In Kamijima, the domestic cats that were positive for FcaGHV1 were mostly found in the western part (Kamiagata) of the island (Figure 1).

### 3.2. Variations of the FcaGHV1 Sequence

Partial *gB* sequences (553 nucleotides) were used for analysis of sequence variations. Sequences from the 31 FcaGHV1-positive animals in this study (three TLCs and 28 domestic cats) contained nucleotide polymorphisms (NPs) at three different positions: 126, 323 and 420. The three TLC sequences were identical to published FcaGHV1 sequences (GenBank KF840715) at nucleotide 126 but with thymidine and adenine replacing cytosine and guanine at nucleotides 323 and 420, respectively. Furthermore, the three TLC sequences were 100% identical to 13 of the 28 sequences obtained from the domestic cats in our study. Similarly, in nine of the 28 domestic cat sequences thymidine replaced cytosine at nucleotide 126 of the published FcaGHV1 (KF840715) sequence. A similar change was present at nucleotide 323 in 13 of the 28 domestic cat sequences and in 17 of the 28 domestic cat sequences adenine replaced guanine at nucleotide 420. Overall, of the 31 sequences obtained in our study, 11 were 100% identical to FcaGHV1 *gB*, KF840715.

Using DNA polymerase gene amplification, we successfully confirmed the presence of FcaGHV1 in all three of the *gB*-positive TLCs. Our PCR detection of FcaGHV1 *DNApol* yielded relatively more non-specific amplification products compared with *gB* PCR. Despite this, we detected FcaGHV1 *DNApol* in 14 of the 28 domestic cats that were positive for the *gB* gene. Sequence analysis of 527 nucleotides revealed 99% homology with GenBank sequences KF840715 and KT595939. Comparison of the nucleotide sequences from the three positive TLCs revealed one nucleotide substitution at position 1219 of KF840715, in which thymidine was replaced by adenine.

### 3.3. Sex and FIV/FeLV Status as Risk Factors for FcaGHV1 Infection in Domestic Cats

Univariable analysis showed that males had a significantly higher probability of being FcaGHV1 positive (odds ratio, 5.86; 95% CI, 2.27–15.14) than females (Table 4). Similar results were obtained following subsequent multivariable analysis (Table 5). Neither FIV nor FeLV infection status was significantly associated with FcaGHV1 detection. The model fit in the multivariable analysis was sufficient (Hosmer–Lemeshow goodness-of-fit *p* = 0.77; *p* > 0.05 suggests an adequate model fit).

### 3.4. Sensitivity of FcaGHV1 gB PCR

**Figure 2:**
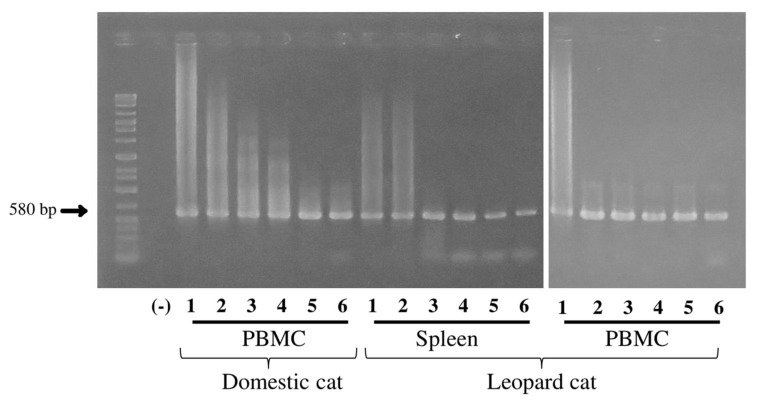
Determination of the sensitivity of FcaGHV1 *gB* PCR. Second-round FcaGHV1 PCR products from PBMCs of a domestic cat and the spleen and PBMCs of a Tsushima leopard cat. bp = base pairs; (-) = FcaGHV1 negative PBMC control; 1 = 1 µg DNA used in first round; 2 = 0.1 µg DNA; 3 = 1 × 10^−2^ µg DNA; 4 = 1 × 10^−3^ µg DNA; 5 = 1 × 10^−4^ µg DNA; 6 = 1 × 10^−5^ µg DNA.

Second-round 580-bp amplicons were amplified from all dilutions (0.01 ng to 1 µg) of spleen and PBMC DNA. The PCR was able to detect virus to at least the 0.01 ng dilution (Figure 2).

### 3.5. Phylogenetic Analyses and Comparison with Other GHVs

**Figure 3:**
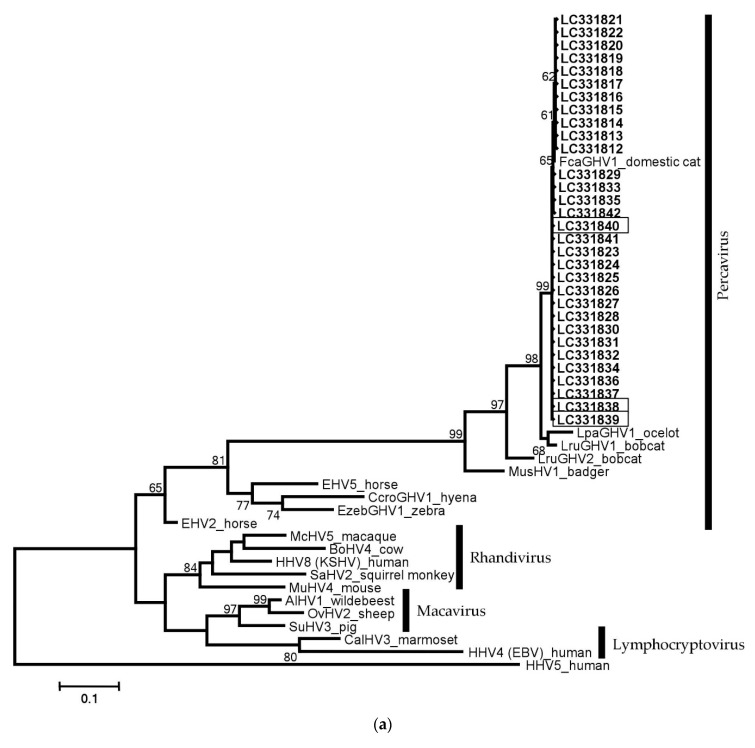

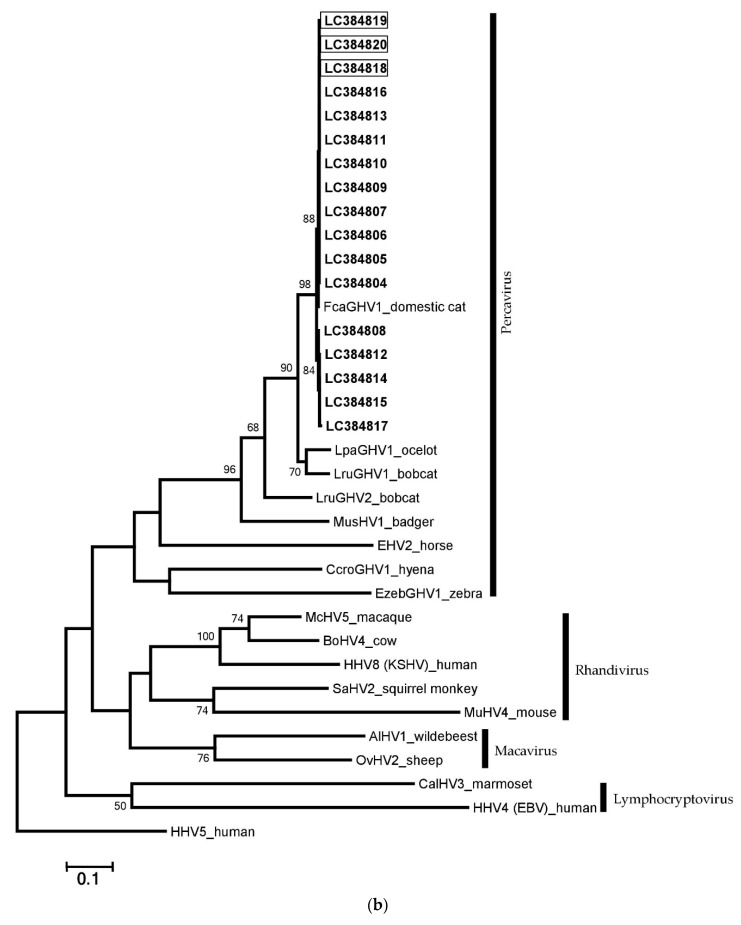
Phylogenetic analysis of gammaherpesviruses (GHVs) using glycoprotein B and DNApol nucleotide alignments. The trees display multiple GHV nucleotide sequences, and betaherpesvirus human cytomegalovirus (human herpesvirus 5; GenBank accession no. NC006273) represents the outgroup. (**a**) The 31 clones of FcaGHV1 *gB* detected in this study are shown in bold (LC331812–LC331842) including three clones isolated from TLCs (indicated with boxes); (**b**) The 17 clones of FcaGHV1 *DNApol* identified in this study are shown in bold (LC384804–LC384820) including three clones isolated from TLCs (indicated with boxes). Maximum-likelihood analysis was performed, and the Kimura 2-parameter model was used to calculate the distance matrix of the aligned sequences, with all branch lengths measured as the number of substitutions per site. Bootstrap support out of 100 replicates is shown for each branch node (values of <50 are not displayed). Virus names, definitions and the GenBank accession numbers of the sequences used in the phylogenetic trees are as follows: Human herpesvirus 4 (HHV4, Epstein–Barr virus, NC007605); Callitrichine herpesvirus 3 (CalHV3, NC004367); Alcelaphine herpesvirus 1 (AlHV1, NC002531); Ovine herpesvirus 2 (OvHV2, NC007646); Mustelid herpesvirus 1 (MusHV1, AF376034); *Felis catus* gammaherpesvirus 1 (FcaGHV1, KF840715); *Lynx rufus* gammaherpesvirus 1 (LruGHV1, KF840716); *Leopardus pardalis* (LpaGHV1, KP721220); *Lynx rufus* gammaherpesvirus 2 (LruGHV2, KP721221); Bovine herpesvirus 4 (BoHV4, NC002665); Saimiriine herpesvirus 2 (SaHV2, NC001350); Suid herpesvirus 3 (AF478169); Equid herpesvirus 2 (EHV2, NC001650); Equid herpesvirus 5 (EHV5, AF050671); *Crocuta crocuta* gammaherpesvirus 1 (CcroGHV1, DQ789371); *Equus zebra* gammaherpesvirus 1 (EzebGHV1, AY495965); Macacine herpesvirus 5 (McHV5, NC003401); Human herpesvirus 8 (HHV8, Kaposi’s sarcoma-associated herpesvirus, NC009333) and Murid herpesvirus 4 (MuHV4, NC001826).

We aligned the FcaGHV1 partial *gB* and *DNApol* sequences to sequences of previously reported viruses for phylogenetic analysis. All FcaGHV1 sequence data detected in the present study formed one cluster with other GHVs within the Percavirus genus (Figure 3a,b).

### 3.6. Nucleotide Sequence Accession Numbers

The partial FcaGHV1 nucleotide sequences obtained in this study have been deposited in the DDBJ, EMBL and GenBank databases under accession numbers LC331812–LC331842 for *gB* and LC384804–LC384820 for *DNApol*.

## 4. Discussion

This is the first study to report the detection of FcaGHV1 DNA in TLCs (*Prionailurus bengalensis euptilurus*). Detection of FcaGHV1 DNA in felids other than the domestic cat—its natural host—suggest that FcaGHV1 can be transmitted to other feline species. A qPCR assay was the first diagnostic tool for FcaGHV1 DNA detection since the discovery of the virus in domestic cats [11]. Even though the sensitivity of qPCR has not yet been fully evaluated and it is thought to usually underestimate the true prevalence of FcaGHV1 infection [14], the majority of previous studies have used this assay. However, the recent development of new indirect ELISAs has enabled not only rapid diagnosis of FcaGHV1 infection but also more sensitive detection of viral exposure [20].

FcaGHV1 DNA detection in TLCs and domestic cats on Tsushima Island provided additional data on the GHV distribution in Japan. Recently, a similar molecular epidemiological study was conducted in Japan to investigate the prevalence and risk factors of FcaGHV1 infection in domestic cats [16]. We decided to extend and include Tsushima Island in this survey of GHV infection because TLCs only live in this region and evidence of FcaGHV1 transmission from domestic cats to TLCs may affect the management strategies that are in place to protect this endangered species.

The frequency of FcaGHV1 detection in Tsushima domestic cats (13.02%) was similar to previously reported frequencies of between 9.6% and 23% in Singapore, Australia, Central Europe (Germany and Austria), the USA and Brazil [11,14,15,17], and higher than the prevalence (1.3%) reported in other parts of Japan [16]. The prevalence and risk of FcaGHV1 infection varies among countries and geographical locations [11,20]. Even though there was no significant difference between the frequency of FcaGHV1 infection in Kamijima and Shimojima, FcaGHV1-positive domestic cats were predominantly found in the western zone of the island.

Being a male was found to be a strongly significant risk factor for FcaGHV1 infection—this supports the previous evidence that FcaGHV1 transmission corresponds with increased aggressive encounters [14,17,20,21].

Our observation that FeLV infection was not associated with an increased likelihood of FcaGHV1 DNA detection is congruent with previous reports [14,16,17,19] but contrasts with specific findings from Singapore where FeLV infection was significantly associated with FcaGHV1 detection [14]. On Tsushima Island, the prevalence rates of FeLV infection in Kamijima and Shimojima were 3.1% and 16.2%, respectively [6]. However, data from epidemiological survey of feline viruses in Singapore, and of particular interest FeLV, were inconsistent [19]. Furthermore, it has been established that FeLV is primarily transmitted through non-aggressive interactions or vertical transmission, in contrast to the tendency of FcaGHV1 to be transmitted through fighting or territorial aggression [14,15,19,21,25]. Additionally, regional differences, different methodological approaches, and the characteristics of the study populations may account for the observed disparity between our results and those found in Singapore.

We found no significant association between increased FcaGHV1 detection and FIV infection. However, in most previous studies the probability of FcaGHV1 detection was significantly higher in FIV-positive cats than in FIV-negative cats and FcaGHV1 viral loads were significantly higher in FIV-infected than non-infected cats [14,15,17,19]. A possible explanation for this difference between our results and those of others is the different methodological approaches and the characteristics of the study population. For example, previous reports were based on well-defined age- and sex-matched control populations [14,15,19] contrary to the current study where only data on individuals’ sex were available. While the detection of FIV provirus DNA was conducted by qPCR [15,17], we employed a serological FIV antibody test. In Singapore, where a large street cat population dominates and lives in free-roaming colonies, the possibility of FIV infection is likely to be high as the virus is spread through biting and fighting [14]. The characteristics of samples recruited for investigation also influence the results. For example, in Brazil, about 50% of FIV-positive cats were positive for FcaGHV1 due to the fact that all samples included in the study were collected from domestic cats with suspected infectious disease [17].

Comparison of the FcaGHV1 *gB* sequences in the 31 isolates obtained in this study with previously reported strains, detected nucleotide variations at positions 126, 323 and 420. Similarly, in Singapore, all FcaGHV1 *gB* isolates had thymidine in place of cytosine at nucleotide 126 of the KF840715 sequence [14]. In another study, one out of six Austrian isolates had guanine in place of adenine at nucleotide position 249 of the KF840715 sequence [15]. In the current study, 11 of 31 isolates were 100% identical to KF840715. Furthermore, previous studies have found that the FcaGHV1 partial *gB* sequences of 12 German, five Austrian, 10 Australian and 23 Japanese isolates were also identical to those found in the USA. This indicates that the FcaGHV1 *gB* gene is relatively conserved despite a few nucleotide polymorphisms.

The isolation of FcaGHV1 from TLCs is important not only to the welfare and health management of TLCs but also because it indicates that the virus may infect feline species other than its natural host. Close interactions between free-roaming domestic cats and TLCs have led to the interspecies transmission of several diseases including parasites and FIV [3,5]. These reports indicate that domestic cats play an important role in disease transmission to TLCs. Even though we have not found any TLCs positive for FeLV, we have demonstrated that the virus can replicate in the cells of TLCs, which suggests that cross-species transmission is possible [6]. The higher prevalence of FcaGHV1 in Tsushima domestic cats than TLCs, and the location of the virus in both TLCs and domestic cats within the same genetic cluster, suggests that transmission from domestic cats to TLCs is likely.

This study provides additional epidemiological information about the FcaGHV1 infection status of feline populations on Tsushima Island. The isolation of FcaGHV1 DNA from TLCs suggests that this disease is an additional risk factor for the health and survival of TLCs.

FcaGHV1 DNA was successfully detected in clinical samples using a virus-specific PCR system targeting the *glycoprotein B* and *DNA polymerase* genes. More FcaGHV1-positive samples were detected by PCR targeting *gB* than by PCR targeting *DNApol*. Furthermore, PCR amplification of *DNApol* also yielded more non-specific amplification products than the *gB* PCR. This limitation should be taken into account when this PCR system is used, and amplicon sequencing is necessary to confirm amplification of the target product.

GHVs are host-specific. Detection of FcaGHV1 DNA in TLCs indicates that the virus is circulating in TLCs. Further studies of FcaGHV1, including the genetic diversity of this virus and its pathogenic potential in other feline species, are needed. In addition, as it has been previously reported that felids can be infected with more than one GHV, future studies to identify other GHVs in TLC populations are warranted.

## Figures and Tables

**Table 1:** Primers for amplification of *Felis catus* gammaherpesvirus 1 *glycoprotein B* and the DNA polymerase.

Name of Primer ^a^	Sequence (5′ to 3′)	Target Gene	Product Size (bp)	Reference
FcaGHV1gB–1s	GACCTGCACCAGAGCATGAG			
FcaGHV1gB–1as	AGGATCCCTGGCAGATTGGT		715	
FcaGHV1gB–2s	TGCACCAGAGCATGAGAGTT			
FcaGHV1gB–2as	TCCCCCGAGAGGGTTTTTGA	*gB*	580	This study
FcaGHV1pol–1s	GGTGTTAATGGAAGCCCTGTG			
FcaGHV1pol–1as	TTAGTCAGCCTTGGCATTGC		818	
FcaGHV1pol–2s	ATGGAAGCCCTGTGAAGTTT			
FcaGHV1pol–2as	CAGTGTCTCATTGCTTGCTGT	*DNApol*	568	This study

^a^ s = sense; as = antisense.

**Table 2:** *Felis catus* gammaherpesvirus 1 *glycoprotein B* detected in feline DNA samples by nested FcaGHV1 PCR.

Host Species	No. of Samples	No. of FcaGHV1 Positive	%Positive (95% CI)
Leopard cat	89	3	3.37 (0.70–9.54)
Domestic cat	215	28	13.02 (8.83–18.27)

**Table 3:** Contingency table of categorical variables with the outcome FcaGHV1 *gB* detection in domestic cats.

Variables	Categories	FcaGHV1 Status		Total	% Positive
		Positive	Negative		
	Male	22	72	94	23
Sex	Female	6	115	121	5
	Kamijima	22	145	167	13
Region	Shimojima	6	42	48	12
	Negative	11	107	118	9
FIV infection	Positive	17	80	97	17
	Negative	22	165	187	12
FeLV infection ^1^	Positive	6	22	28	21

^1^ Cited from reference number 6.

**Table 4:** Univariable logistic regression analyses to evaluate the association between explanatory variables and the outcome, FcaGHV1 detection in domestic cats.

Variables	Categories	b ^1^	SE ^2^	Odds-Ratio	95% CI	*p*-Value
Intercept	Male vs. female	−0.58	0.64			
Sex		1.77	0.48	5.86	2.27–15.14	<0.0001
Intercept	Kamijima vs. Shimojima	2.13	1.87			
Region		−0.06	0.49	0.94	0.36–2.47	0.903
Intercept	Positive vs. negative	−2.08	2.25			
FIV infection		0.73	0.41	2.07	0.92–4.66	0.080
Intercept	Positive vs. negative	−3.71	4.01			
FeLV infection		0.72	0.51	2.05	0.75–5.60	0.163

^1^ Coefficient of variable estimate; ^2^ Standard error of the variable estimate; SE, Standard error; CI, Confidence interval.

**Table 5:** Multivariable logistic regression analyses to identify risk factors for FcaGHV1 detection in domestic cats.

Variables	Categories	b ^1^	SE ^2^	Odds-Ratio	95% CI	*p*-Value
Intercept		−11.02	5.87			
Sex	Male vs. female	1.64	0.49	5.17	1.95–13.70	0.001
FIV infection	Positive vs. negative	0.57	0.47	1.77	0.71–4.44	0.223
FeLV infection	Positive vs. negative	0.96	0.58	2.60	0.84–8.09	0.099

^1^ Coefficient of variable estimate; ^2^ Standard error of the variable estimate; SE, Standard error; CI, Confidence interval.
